# Diagnosis and Treatment of Chronic Neuropathic and Mixed Pain in Children and Adolescents: Results of a Survey Study amongst Practitioners

**DOI:** 10.3390/children7110208

**Published:** 2020-11-02

**Authors:** Thomas G. de Leeuw, Tjitske van der Zanden, Simona Ravera, Mariagrazia Felisi, Donato Bonifazi, Dick Tibboel, Adriana Ceci, Florentia Kaguelidou, Saskia N. de Wildt, on behalf of the GAPP Consortium

**Affiliations:** 1 Department of Pediatric Anesthesia and Pain Medicine, Erasmus MC-Sophia Children’s Hospital, Dr. Molewaterplein 40, 3015 GD Rotterdam, The Netherlands; 2 Intensive Care and Pediatric Surgery, Erasmus MC-Sophia Children’s Hospital, Dr. Molewaterplein 40, 3015 GD Rotterdam, The Netherlands; T.vanderZanden@ErasmusMC.nl (T.v.d.Z.); D.Tibboel@ErasmusMC.nl (D.T.); Saskia.deWildt@Radboudumc.nl (S.N.d.W.); 3 Pharmaceutical Research Management Srl, Via Luigi Porta 14, 27100 Pavia, Italy; Trialsupport@pharmsrl.com (S.R.); Trialsupport@pharmsrl.it (M.F.); 4 Consorzio per Valutazioni Biologiche e Farmacologiche, Via Putignani 178, 70122 Bari, Italy; DonatoBonifazi@cvbf.net; 5 Fondazione per la Ricerca Farmacologica Gianni Benzi onlus, Via Abate Eustasio 30, 70010 Valenzano, Italy; AdriCeci.uni@Gmail.com; 6 Centre d’Investigations Cliniques, INSERM CIC1426, Hôpital Robert Debré, APHP, Université de Paris, UMR-1123 ECEVE, 75019 Paris, France; Florentia.Kaguelidou@aphp.fr; 7 Department of Pharmacology and Toxicology, Radboud Institute Health Sciences, Radboud University Medical Center, Geert Grooteplein Zuid 10, 6525 GA Nijmegen, The Netherlands

**Keywords:** chronic, neuropathic pain, mixed pain, children, diagnosis, treatment, practitioners

## Abstract

Validated diagnostic tools to diagnose chronic neuropathic and mixed pain in children are missing. Therapeutic options are often derived from therapeutics for adults. To investigate the international practice amongst practitioners for the diagnosis and treatment of chronic, neuropathic pain in children and adolescents, we performed a survey study among members of learned societies or groups whose members are known to treat pediatric pain. The survey included questions concerning practitioners and practice characteristics, assessment and diagnosis, treatment and medication. We analyzed 117 returned questionnaires, of which 41 (35%) were fully completed and 76 (65%) were partially completed. Most respondents based the diagnosis of neuropathic pain on physical examination (68 (58.1%)), patient history (67 (57.3%)), and underlying disease (59 (50.4%)) combined. Gabapentin, amitriptyline, and pregabalin were the first-choice treatments for moderate neuropathic pain. Tramadol, ibuprofen, amitriptyline, and paracetamol were the first-choice treatments for moderate mixed pain. Consensus on the diagnostic process of neuropathic pain in children and adolescents is lacking. Drug treatment varies widely for moderate, severe neuropathic, and mixed pain. Hence, diagnostic tools and therapy need to be harmonized and validated for use in children.

## 1. Introduction

The diagnosis and treatment of chronic neuropathic and mixed pain in children and adolescents is very challenging for various reasons. First, the diagnostic criteria for neuropathic pain advised by the International Association for the Study of Pain (IASP) [1], such as specific diagnostic tools, including questionnaires, quantitative sensory testing (QST), or electromyography (EMG), have not been validated or cannot be easily applied to children, as underlying pathology is often different from that in adults. For example, neuropathic pain caused by diabetic neuropathy, radiculopathy, and strokes is rare in children, whereas congenital and autoimmune disorders and metabolic diseases in childhood as causes for neuropathic pain are hardly seen in the adult population [2,3]. Second, children with chronic pain are not only treated by pediatric pain specialists but also by other practitioners who might not consider the possibility of a neuropathic origin of the pain. Therefore, a considerable delay in diagnosis cannot be excluded [4]. It is important to consider the possibility of neuropathic pain or a neuropathic component in mixed pain, since the treatment is quite different from that for nociceptive or inflammatory pain. Severe and long-lasting pain problems in childhood might result in persistence of pain or development of other chronic pain states in adulthood [5,6].

The evidence for pharmacologic treatment of chronic neuropathic pain in children has mainly been derived from research in adults. Specific pediatric evidence is limited to case reports or is completely lacking [7]. The use of gabapentin in the management of chronic pain with neuropathic characteristics is one of the top-three research priorities in the field of pediatric pain [8]. In daily practice, compliance to medication therapy (specifically to gabapentinoids) by patients and parents of younger children is often suboptimal, because adverse effects, such as dizziness and vision disturbances, may occur.

Hence, while pediatric pain is prevalent, evidence-based guidance for physicians to diagnose and treat children is lacking. Moreover, as registered drugs to treat chronic pain in children are scarce, physicians are left to perform their own benefit-risk analysis when prescribing off-label drugs.

We therefore performed a survey study to evaluate the current international practice of diagnosis and treatment of chronic neuropathic and mixed pain in children. The results were expected to aid shaping the research agenda, designing clinical drug trials and developing practice guidelines.

This study was conducted as part of the GAbapentin in Paediatric Pain (GAPP)-project, a EU-funded development plan for gabapentin in children, with the purpose of aiding the design of two clinical trials to be conducted in the context of this plan.

## 2. Methods

### 2.1. Study Design

A survey study, for which we developed a web-based questionnaire covering practitioner characteristics, diagnostic procedures, and pharmacotherapy, aimed at the international pediatric pain community.

### 2.2. Questionnaire Development

The questionnaire was developed by a pediatric anesthesiologist-pain specialist (T.d.L.), a pediatric intensivist-clinical pharmacologist (S.N.W.), a project manager (T.Z.), and a pediatrician-clinical pharmacologist (F.K.), all of them GAPP consortium partners. The first version of the questionnaire was distributed among partners of the GAPP consortium and revised after feedback had been received. The content of the second version was then approved by the GAPP-consortium.

The questionnaire consisted of 33 questions divided in 3 sections (see Supplementary material).

#### 2.2.1. Treatment Setting

Age, sex, profession, work place, type of hospital, type of clinic, presence of a dedicated pain team, involvement of different specialists in the treatment, percentage of working week spent treating children with chronic pain, number of new patients seen in one year, and frequency of clinical visits.

#### 2.2.2. Diagnostic Assessment

Diagnosis, use of specific pain questionnaires and scales, use of physical functioning or quality of life scale (PedsQl) [9], use of diagnostic IASP criteria [1], use of additional diagnostics, and presence of underlying clinical conditions.

#### 2.2.3. Treatment

Presence of standardized protocols for neuropathic or mixed pain management, first line treatment (1st choice, 2nd choice), and second line treatment (switch from first line or add-on) for moderate (numeric rating scale (NRS) ≥ 4 and < 7) and for severe (NRS > 7) neuropathic and mixed pain, use of titration schedules for different drugs, use of gabapentin and its starting, target, and maximum doses and duration of treatment, and non-pharmacological approaches.

An online questionnaire was built using Survey Monkey (Survey Monkey Inc., San Mateo, CA, USA). Items could be left blank upon the respondent’s discretion. Members of the European Society for Paediatric Anaesthesiology (ESPA), the IASP Special Interest Group on Pain in Children, and the Pediatric Pain List received an invitation by email with a link to the questionnaire. These groups totaled approximately 1400 individuals, including duplicate memberships and a relatively large proportion of pediatric anesthesiologists (ESPA members) not directly involved in the treatment of chronic pain. One reminder to complete the questionnaire was sent to these lists.

According to the Dutch Medical Research Involving Human Subjects Act, neither ethical approval nor informed consent was needed. Data were collected between July 2016 and February 2017.

### 2.3. Data Analysis

Data were analyzed using standard statistics in SPSS Statistics version 24 (IBM, Armonk, NY, USA).

## 3. Results

A total of 117 questionnaires were returned, of which 41 (35%) were fully completed and 76 (65%) partially completed. Analyses were performed on all 117 returned questionnaires.

### 3.1. Treatment Setting

A total of 40 respondents were from Europe, 54 were from the USA/Canada, 5 were from Australia/New Zealand, 7 were from Asia, and 4 were from Africa. Seven respondents were from unknown origin.

Overall, most respondents were pediatric anesthesiologists, pediatric anesthesiologists-pain specialists, or specialized nurses (Table 1a). Pediatric anesthesiologists and pediatricians are mostly involved in the treatment of chronic pain in clinics of the respondents, followed by pediatric oncologists, pediatric neurologists, and rehabilitation specialists (Table 1b). Almost half of the practitioners (48.7%) spent less than 30% of their working week treating children with chronic pain (Table 2).

More than half of respondents were based in either a university or a general children’s hospital (56%). Almost one third (29%) were based in a general or university hospital. Almost one tenth (9%) were based in a public hospital, and only few were based in a private hospital, rehabilitations clinic, or otherwise (2% each). More than half (63%) of the respondents reported the availability of a dedicated pain team.

### 3.2. Chronic and Neuropathic Pain Diagnosis

#### 3.2.1. Chronic Pain Definition

A total of 47 (40.2%) of the respondents used the following definition for the diagnosis of chronic pain: “Pain persisting past time of normal healing”; 31 (26.5%) used: “Pain lasting more than 3 months”; 10 (8.5%) used another definition, of whom 4 (3.4%) used both previous definitions; 29 (24.8%) left this item unanswered.

#### 3.2.2. Diagnosis

A total of 53 (45.3%) of the respondents used the IASP criteria for establishing the diagnosis of neuropathic pain (“pain initiated or caused by a primary lesion or dysfunction in the nervous system”), while 18 (15.4%) explicitly answered not to use these criteria; 46 persons (39.3%) left this question unanswered. Most practitioners based the diagnosis of neuropathic pain on a combination of physical examination, history, and/or underlying disease. Diagnostic tools play a limited role in diagnosing neuropathic pain (Table 3).

A total of 36 (30.8%) of the respondents used a specific questionnaire to diagnose neuropathic pain; 8 (6.8%) of them used more than one. The questionnaires/scales used were the Neuropathic Pain Questionnaire (NPQ) (10.3%) [10], ‘Douleur Neuropathique en 4 questions’ (DN4) (7.7%) [11], Pain Detect (6.0%) [12], LANSS Leeds Assessment of Neuropathic Symptoms and Signs (5.1%) [13], or ID-Pain (1.7%) [14]. A total of 45 (38.5%) used none of these and 44 (37.6%) left this question unanswered.

A total of 27 (23.1%) respondents did not use additional diagnostic tools. Several diagnostic tools, in addition to physical examination and pain scores, were used (more than one option was possible). Magnetic resonance imaging (MRI) was used by 30 (25.6%), Electromyography (EMG) was used by 28 (23.9%), quantitative sensory testing (QST) was used by 23 (19.7%), and Nerve biopsy was used by 3 (2.6%) practitioners; 26 (22.2%) respondents used more than one additional tool.

Practitioners (N = 117) annually consulted on average on 5 (0–50) patients with chronic neuropathic or mixed pain between 3 months and 3 years of age, 17 (0–80) patients from 3 years up to 8 years of age, and 104 (0–1100) patience from 8 till 18 years of age.

The estimated distribution of neuropathic and mixed pain in patients seen by respondents was quite similar for the three age groups: approximately 25–35% versus 60–66%.

The most frequently reported underlying clinical conditions, as a cause for neuropathic or mixed pain, were trauma and postoperative neuropathic pain (48.7%), complex regional pain syndrome (45.3%), and cancer (treatment) (38.5%). Many other underlying diseases were reported, though less frequently (Table 4)**.** A total of 66 (56.4%) respondents indicated more than one condition as a cause for the neuropathic pain; 48 (41%) left the question unanswered.

### 3.3. Pharmacological Treatment

Only 11 respondents (9.4%) reported to use a standard protocol for the management of neuropathic pain; 42 respondents (35.9%) did not use a protocol or had no standard protocol; 64 (54.7%) left this question unanswered.

#### 3.3.1. Neuropathic Pain

For moderate neuropathic pain (NRS ≥ 4 but < 7), the most frequently used drugs as first-line treatments are gabapentin, amitriptyline, and pregabalin (top 3); the top 3 add-ons to first-line drugs consists of amitriptyline, pregabalin, and gabapentin (Table 5a,b). For severe neuropathic pain (NRS > 7), the most frequently used drugs as first-line treatments are gabapentin, amitriptyline, and pregabalin; the top 3 of add-ons to first-line drugs are gabapentin, amitriptyline, and ketamine (Table 5c,d).

#### 3.3.2. Mixed Pain

For moderate mixed pain, the top 3 first-line drugs consists of tramadol, ibuprofen, and amitriptyline; gabapentin, pregabalin, and tramadol are most frequently used as add-ons (Table 6a,b). Severe mixed pain is primarily treated with amitriptyline, morphine, and tramadol; with gabapentin, morphine, and amitriptyline as add-ons (Table 6c,d).

#### 3.3.3. Dose Titration

The respondents mainly used titration schedules for antiepileptics, antidepressants, and opioids (Table 7). One respondent described tapering off opioids, gabapentin, and amitriptyline. Most respondents started titrating gabapentin with doses between 5 to 10 mg/kg/day (29/117 = 24.7%). The preferred maintenance dose was 30 mg/kg/day but was generally between 10 to 30 mg/kg/day (31/117 = 26.5%). The preferred maximum dose was 60 mg/kg/day but was generally between 30 to 60 mg/kg/day (27/117 = 23.1%) (Table 8). A total of 29 (24.7%) respondents waited between 2 and 6 (range 1–20 weeks) weeks before adding medication to gabapentin if gabapentin alone was not effective. On the contrary, 32 (27.4%) reduced or stopped gabapentin between 2 to 8 weeks (range 1–30 weeks) if it appeared not to be effective, while 44 (37.6%) respondents agreed to change first-line medication in in the context of a research protocol, though 5 mentioned it would not be feasible in their practices. A total of 6 respondents stated that they would not change their first-line medication; 67 left this question unanswered.

### 3.4. Non-Pharmacological Therapy

A total of 51 (44%) of the respondents confirmed the use of non-pharmacologic approaches; 66 (56%) left the question unanswered. Physiotherapy and cognitive behavioral therapy were the most used approaches (Table 9).

## 4. Discussion

The results of this survey study give insight into the international practice amongst practitioners dealing with neuropathic and mixed chronic pain in children. It appears that specialists of various disciplines are involved in the treatment of chronic pain in children. There is a fairly large variation in the way practitioners establish the diagnosis of chronic neuropathic pain. Interestingly, there is more consensus in the choice of medication used to treat chronic neuropathic pain in children, although very few practitioners use a standard protocol and none of the drugs prescribed have been registered for use in children for the treatment of pain. Almost all practitioners use different non-pharmacologic approaches.

### 4.1. Diagnosis

There is no absolute consensus on the definition of chronic pediatric pain or on the criteria for neuropathic pain in children. Experimental neurophysiology even suggests that neuropathic pain following neurologic injury is absent in young children due to a change of microglial response during development in childhood, whereby nerve injury in early childhood only emerges as neuropathic pain during adolescence [15]. Nevertheless, neuropathic pain has been reported even in very young children due to, e.g., metabolic disorders, cancer, chemotherapy, neuromuscular disease, operations, or traumatic injuries. Neuropathic pain in children can be difficult to diagnose [2], especially in younger children who might find it difficult to express the character of their pain.

Around half of the respondents use the IASP criteria [1] for establishing the diagnosis of neuropathic pain. Others explicitly stated not to use them on children. Therefore, specific validation of these criteria for children is highly recommended.

For establishing the diagnosis of neuropathic pain, most respondents stated that they would use physical examination, patient history, and possible underlying disease combined. Around one third used a specific questionnaire, mostly the Neuropathic Pain Questionnaire (NPQ) [10], Douleur Neuropathique 4 (DN4) [11], or Pain Detect [12]. As described in the literature, the DN4 and NPQ seem to be most suitable for clinical use, although they have limited value compared to thorough clinical assessment [16]. None of these questionnaires have been validated for children.

Additional diagnostic tools were also rarely used: MRI by 25% of respondents; the quite painful, and therefore less suitable, EMG by around 25% of respondents; and QST by 20% of respondents. QST provides an opportunity for improvement of the diagnostic process, as it also seems most promising for the prediction of response to treatment. However, its use requires a trained staff next to equipment [17].

The somewhat strict classification by the IASP, either nociceptive or neuropathic pain, leaves out a considerable number of children presenting with symptoms of both; therefore, it is referred to as “mixed pain” by clinicians. As discussed in a recent review, the use of this concept can have therapeutic consequences [18]. The distribution between neuropathic and mixed pain cases in daily practice is around one-third versus two-thirds, as estimated by respondents in our survey. Trauma and operation are most often regarded here as the cause of neuropathic and/or mixed pain in children, followed by complex regional pain syndrome, cancer related pain, and phantom limb pain.

### 4.2. Treatment

A biopsychosocial, interdisciplinary approach for the treatment of chronic pain in children is strongly advised [19,20]. Despite the evidence for this approach, treatment outside pediatric pain centers still varies widely. Although physiotherapists and psychologists are not always involved in the treatment of chronic pain, practitioners who completed the questionnaire almost all used non-pharmacologic approaches next to medication, where physiotherapy, cognitive behavioral therapy, and transcutaneous electric nerve stimulation were mentioned most.

One could argue that a non-pharmacologic approach with physiotherapy and psychologic support should be started previous to or next to medication in any case of neuropathic pain in children [20,21]. In case of a localized pain problem, a lidocaine patch or a non-invasive technique without side effects, such as transcutaneous electrical nerve stimulation, could be considered, although evidence for these therapies is limited [21,22,23].

### 4.3. Medication

A recent systematic review on pharmacologic interventions for chronic pain in children showed that the overall quality of evidence was very low [24]. In another recently published systematic review, five of the seven studies dealt with the use of gabapentin as a prophylactic pain relief and just two studies concerned the use of gabapentin and pregabalin in pediatric chronic pain states (complex regional pain syndrome and fibromyalgia). No significant advantage of gabapentin over amitriptyline (gabapentin study) or placebo (pregabalin study) was found, but both chronic pain studies had methodological shortcomings [25].

Nevertheless, the reported drug treatment of moderate neuropathic pain and severe neuropathic pain in this survey was quite uniform, although merely less than 10% of practitioners used a standardized protocol for pharmacological treatment of neuropathic pain. An often-used strategy is starting treatment with gabapentin or pregabalin and titrating up to the recommended maintenance dose, after which amitriptyline is added in case of insufficient effect or additional sleeping problems. This strategy is most likely derived from therapeutic recommendations for adults [26], as the use for neuropathic pain in children of these drugs is off-label and guidelines are lacking. In adult studies, the use of tricyclic antidepressants, gabapentin, and pregabalin is highly recommended for several neuropathic pain conditions, with lower numbers needed to treat (NNT) for tricyclic antidepressants than for gabapentin and pregabalin, respectively [26]. Since the recommendations for use in children are not very clear—lacking formalized dosages, just dosages derived from adult practice—the variety in prescribing practice is not surprising. For this reason, especially younger children could be undertreated. Ouellet et al. advised in 2001 that based on creatinine clearance and volume of distribution, children younger than 5 years treated with gabapentin for epilepsy should receive a 33% larger dose to achieve the same exposure as older children [27]. As no major differences in pharmacokinetics are to be expected between children with epilepsy or neuropathic pain, this advice is likely also applicable in the treatment of pain.

Therefore, more research is urgently needed with recommendations for the right starting dosages, maintenance dosages, and maximum dosage to guarantee an adequate medication plasma level in the different pediatric age groups [26]. Anghelescu et al. published an algorithm for the treatment of neuropathic pain in pediatric oncology [28], an algorithm for other causes of neuropathic pain in children and treatment in outward patient setting should be developed likewise.

Regarding adverse effects, such as unintentional overdose and suicidal ideation at adolescent age, pregabalin is associated with a higher risk of adverse effects than gabapentin. Therefore, gabapentin appears to be a safer option for use in children and adolescents [29].

An exception to the adult practice is the frequent add-on of ketamine for severe neuropathic pain. Ketamine, as a *N*-methyl-d-aspartate antagonist, has a modulating effect on ascending nociceptive transmission, as well as on descending inhibitory pathways [30]. A disadvantage is that its use is limited to a clinical setting.

For moderate mixed chronic pain, tramadol appears to be the first medicine of choice, together with non-steroid-anti-inflammatory drugs (NSAIDs), amitriptyline. or paracetamol, with more specific anti-neuropathic medication as an add–on, such as gabapentin, pregabalin, or tramadol. For severe mixed chronic pain, the same drugs are used, together with morphine, as the first drug of choice or as an add-on.

Tramadol is most often used, presumably because of the double action on the µ-opioid-receptor and as a serotonin- and noradrenalin-uptake inhibitor [31]. Although tramadol is also very suitable as rescue medication and, in droplets, is easy to dose even to very young children, its metabolite 0-desmethyl-tramadol has a 200-fold higher affinity for the µ-receptor, which can cause respiratory depression in ultra-rapid metabolizers. This has led to warnings of USA Food and Drug Administration (FDA) and the European Society for Paediatric Anaesthesiology (ESPA). In the USA, tramadol is therefore contraindicated < 12 years for all children and < 18 years in ear, nose and throat-surgery. In Europe, its use is recommended only for acute postoperative pain in a monitored setting [32]. Tramadol might be related to a greater risk of overdose than oxycodone when used by adolescents [33].

Tapentadol, a recently released drug with a similar mechanism of action as tramadol, is still sparsely used in chronic mixed pediatric pain. The lack of an active metabolite may make it a safer option for use in children. On the other hand, in adults its use appears to cause more serious adverse effects than tramadol [34]. Though not yet officially registered for use in children, first evaluations of pharmacokinetic, safety, and efficacy results have been published, which show a good tolerability and safety in children from 6 to 18 years when used for acute pain, with the same plasma concentrations as those reached in adults [35,36].

In case of severe mixed pain, opioids, such as morphine, oxycodone, and tramadol, appear to be frequently used. The use of opioids in chronic non-cancer pain in children is debated. The general point of view is that their use is not of great value and even might have potential negative outcomes, because adverse drug reactions, such as constipation, cognitive dysfunction, or psychiatric comorbidities, and also tolerance or opioid-induced hyperalgesia, might prevail above the analgesic effect. Nonetheless, a review found no evidence from randomized controlled trials to support or refute the use of opioids to treat chronic non-cancer pain in children and adolescents [37].

Cyclooxygenase-2 (COX-2) inhibitors are hardly used for mixed pain conditions in children, despite their presumed better safety profile compared to classical NSAIDs. A Cochrane review identified only a small number of studies, with insufficient data for analysis for the use of NSAIDs in chronic non-cancer pain. The limited evidence comes from adult studies [38].

### 4.4. Practitioners

A variety of specialists appear to be involved in the treatment of children with neuropathic pain, of whom the majority are pediatric anesthesiologists and pediatricians. Although most of the children are treated in dedicated pediatric centers, almost half of the professionals do not spend more than 30% of their time dealing with chronic pain in children or do not work in a dedicated Pediatric Pain Center. One could debate, considering the complexity of chronic pediatric pain, that all chronic pediatric pain treatment should preferably take place in a Pediatric Pain Center [4,39].

## 5. Limitations of the Study

To our knowledge, this is the first international survey that focuses on how practitioners diagnose and treat chronic neuropathic pain in children and adolescents.

While we reached out to approximately 1400 professionals, only 117 contributions were received, of which just 41 (35%) were complete. We learned through informal feedback that the low response rate was likely due to the large number of detailed questions. Some respondents argued that the questionnaire should have been more limited in order to be really thorough. This shows the limitations of using questionnaires to understand current practice with the problem of finding the right balance between being thorough without being over-detailed. Hence, our data should be interpreted qualitatively and not quantitatively.

Furthermore, the questionnaire was only distributed among members of the European Society of Pediatric Anesthesia, members of the Special Interest Group on Pain in Children from the IASP, and practitioners who have a subscription to the Pediatric Pain list. Therefore, there might be a relatively high proportion of anesthesiologists among the respondents, although membership of SIG on Pain in Children from the IASP and the Pediatric Pain List is accessible for everyone professionally involved in treating pain in children.

Some respondents discussed the lists of medications provided, which might be explained by differences in medication available in the USA/Canada and Europe.

## 6. Overall Conclusion/Future Directions

There is a wide array of practice in chronic pediatric pain, not only organizational, but also with respect to diagnosis and treatment. Diagnoses are based on physical examination and history only, since validated pediatric tools are lacking. Treatment in specialized pediatric pain centers is not implemented on a large scale. In the context of the lack of evidence-based data on almost all aspects of treatment, despite apparent consensus on the use of gabapentin and amitriptyline, standardized protocols are needed for the treatment of neuropathic pain in children, also in light of future prospective studies. Guidance for future research to obtain the evidence is given in the literature [8].

## Figures and Tables

**Table 1:** Professions of questionnaire respondents (**a**) and professions of specialists involved in treatment of chronic pain in children in respondents’ hospitals (**b**).

a. Professions of Respondents (*N* = 117) No. (%)		b. Professions of Specialists Involved in the Treatment of Chronic Pain in Children in Clinics of Respondents No.	
Pediatric anesthesiologist-pain specialist	31 (26.5%)	Pediatrician	52
Pediatric anesthesiologist	26 (22.2%)	Pediatric anesthesiologist-Pain specialist	51
Nurse practitioner	15 (12.8%)	Pediatric oncologist	41
Anesthesiologist	13 (11.1%)	Pediatric neurologist	36
Pediatrician	10 (8.5%)	Rehabilitation specialist	26
Nurse	4 (3.4%)	Anesthesiologist	15
General practitioner/family doctor	2 (1.7%)	General practitioner/family doctor	12
Pediatric neurologist	2 (1.7%)	Neurologist	7
Pediatric oncologist	2 (1.7%)	Clinical pharmacologist	7
Pediatrician-pain specialist	2 (1.7%)	Others	33
Pediatrician-palliative care	2 (1.7%)		
Pediatric pain psychologist	2 (1.7%)	Most frequent others specified:	
Anesthesiologist-pain specialist	1 (0.8%)	Clinical psychologist	14
Clinical pharmacologist	1 (0.8%)	Physiotherapist	7
Pain specialist	1 (0.8%)	Nurse and nurse practitioners	7
Pediatric rheumatologist	1 (0.8%)	Pediatric psychiatrist	5
Rehabilitation specialist	1 (0.8%)	Palliative care specialist	5
Researcher	1 (0.8%)	Rheumatologist	5

**Table 2:** Proportion of working week spent on treating children with chronic pain.

Time (%) of Working Week	Total (*N* = 117) No. (%)
≤10%	30 (25.6%)
>10%–≤30%	27 (23.1%)
>50%–≤80%	13 (11.1%)
>30%–≤50%	11 (9.4%)
>80%	2 (1.7%)
No answer	34 (29.1%)

**Table 3:** Condition on which practitioners base the diagnosis of neuropathic pain.

Diagnosis Neuropathic Pain Based On: No. (%) Total *N* = 117 (More Than One Item Applicable)	
Based on physical examination	68 (58.1%)
Based on history	67 (57.3%)
Based on underlying disease	59 (50.4%)
Based on treatment for another disease	26 (22.2%)
Based on diagnostic tests	25 (21.4%)
Other	8 (6.8%)
Including: response to treatment	2 (1.7%)

**Table 4:** Underlying condition for neuropathic/mixed pain.

Underlying Clinical Condition	No. (%)
Trauma and postoperative neuropathic pain	57 (48.7%)
Complex Regional Pain Syndrome	53 (45.3%)
Effects of cancer disease/treatment	45 (38.5%)
Phantom limb pain	32 (27.4%)
Autoimmune, degenerative, or inflammatory neuropathies	24 (20.5%)
(e.g., Guillain–Barré syndrome, multiple sclerosis)	
Spinal cord injury	21 (17.9%)
Burns	17 (14.5%)
Hereditary neurodegenerative disorders	16 (13.7%)
(e.g., Fabry disease)	
Infections	14 (12.0%)

**Table 5:** Prevalence of first (and second) medication of choice and medication used as an add-on in case of moderate and severe neuropathic pain.

a. Neuropathic Moderate (NRS 4–6) Pain Initial Treatment:			b. Neuropathic Moderate (NRS 4–6) Pain Add On:			c. Neuropathic Severe (NRS > 7) Pain Initial Treatment:			d. Neuropathic Severe (NRS > 7) Pain Add On:		
Drug of choice	1st	2nd	Drug of choice	1st	2nd	Drug of choice	1st	2nd	Drug of choice	1st	2nd
Gabapentin	32 (27.4%)	13 (11.1%)	Amitriptyline	18 (15.5%)	6 (5.1%)	Gabapentin	26 (22.2%)	10 (8.5%)	Gabapentin	13 (11.1%)	5 (4.3%)
Amitriptyline	7 (6.0%)	17 (14.5%)	Pregabalin	8 (6.8%)	6 (5.1%)	Amitriptyline	9 (7.7%)	10 (8.5%)	Amitriptyline	9 (7.7%)	3 (2.6%)
Pregabalin	6 (5.1%)	10 (8.5%)	Gabapentin	5 (4.3%)	4 (3.4%)	Pregabalin	5 (4.3%)	8 (6.8%)	Ketamine	6 (5.1%)	2 (1.7%)
Paracetamol	4 (3.4%)		Lidocaine	4 (3.4%)	1 (0.9%)	Ketamine	2 (1.7%)	2 (1.7%)	Pregabalin	4 (3.4%)	10 (8.5%)
Ibuprofen	1 (0.9%)		Paracetamol	2 (1.7%)	1 (0.9%)	Morphine	2 (1.7%)	2 (1.7%)	Tramadol	3 (2.6%)	1 (0.9%)
Nortriptyline	1 (0.9%)	1 (0.9%)	Ibuprofen	2 (1.7%)	1 (0.9%)	Tramadol	1 (0.9%)	3 (2.6%_	Morphine	3 (2.6%)	2 (1.7%)
Lidocaine	1 (0.9%)	1 (0.9%)	Diclofenac	2 (1.7%)	1 (0.9%)	Nortriptyline	1 (0.9%)	2 (1.7%)	Oxycodone	2 (1.7%)	3 (2.6%)
Tramadol	3 (2.6%)		Carbamazepine	1 (0.9%)	3 (2.6%)	Carbamazepine	1 (0.9%)	1 (0.9%)	Lidocaine	2 (1.7%)	2 (1.7%)
Celecoxib	1 (0.9%)		SNRIs	1 (0.9%)	2 (1.7%)	Lidocaine	1 (0.9%)	1 (0.9%)	Other opioids	1 (0.9%)	3 (2.6%)
Etoricoxib	1 (0.9%)		Tramadol	1 (0.9%)	3 (2.6%)	Paracetamol	1 (0.9%)		Naproxen	1 (0.9%)	
Carbamazepine	1 (0.9%)		Oxycodone	1 (0.9%)	2 (1.7%)	Hydromorphone	1 (0.9%)		Diclofenac	1 (0.9%)	
Morphine	1 (0.9%)		Morphine	1 (0.9%)	1 (0.9%)	Oxycodone	2 (1.7%)		Fentanyl	1 (0.9%)	
Other opioids	1 (0.9%)		Hydromorphone	1 (0.9%)		SNRIs	1 (0.9%)		SNRIs	3 (2.6%)	
None o/t above	1 (0.9%)	2 (1.7%)	Other opioids	1 (0.9%)	1 (0.9%)	Other opioids	1 (0.9%)		Nortriptyline	2 (1.7%)	
No Answer	64 (54.7%)	65 (55.6%)	Capsaicin	1 (0.9%)	1 (0.9%)	No alternative	2 (1.7%)		Hydromorphone	1 (0.9%)	
			Nortriptyline	2 (1.7%)		None o/t above	1 (0.9%)	1 (0.9%)	Ketorolac	1 (0.9%)	
			Ketamine	2 (1.7%)		No Answer	66 (56.4%)	71 (60.7%)	Carbamazepine	1 (0.9%)	
			Tapentadol	1 (0.9%)					No alternative	1 (0.9%)	4 (3.4%)
			Fentanyl	1 (0.9%)					None o/t above	1 (0.9%)	1 (0.9%)
			No alternative	3 (2.6%)					No Answer	69 (59.0%)	73 (62.4%)
			None o/t above	2 (1.7%)	2 (1.7%)						
			No Answer	66 (56.4%)	73 (62.4%)						

Abbreviations: serotonin- and noradrenalin-reuptake inhibitors (SNRIs); numeric rating scale (NRS).

**Table 6:** Prevalences of first (and second) medication of choice and medication used as add-on in case of moderate and severe mixed pain.

a. Moderate Mixed (NRS 4–6) Pain Initial Treatment:			b. Moderate Mixed (NRS 4–6) Pain Add On:			c. Severe Mixed (NRS > 7) Pain Initial Treatment:			d. Severe Mixed (NRS > 7) Pain Add On:		
Drug of choice	1st	2nd	Drug of choice	1st	2nd	Drug of choice	1st	2nd	Drug of choice	1st	2nd
Tramadol	10 (8.5%)	7 (6.0%)	Gabapentin	10 (8.5%)	4 (3.4%)	Amitriptyline	11 (9.4%)	5 (4.3%)	Gabapentin	9 (7.7%)	5 (4.3%)
Ibuprofen	8 (6.8%)	8 (6.8%)	Pregabalin	5 (4.3%)	3 (2.6%)	Morphine	9 (7.7%)	3 (2.6%)	Morphine	8 (6.8%)	3 (2.6%)
Amitriptyline	8 (6.8%)	5 (4.3%)	Tramadol	5 (4.3%)	3 (2.6%)	Tramadol	6 (5.1%)	5 (4.3%)	Amitriptyline	7 (6.0%)	3 (2.6%)
Paracetamol	8 (6.8%)	2 (1.7%)	Ibuprofen	5 (4.3%)	1 (0.9%)	Oxycodone	4 (3.4%)	2 (1.7%)	Tramadol	3 (2.6%)	1 (0.9%)
Naproxen	4 (3.4%)		Amitriptyline	4 (3.4%)	2 (1.7%)	Gabapentin	3 (2.6%)	10 (8.5%)	Naproxen	3 (2.6%)	
Gabapentin	3 (2.6%)	12 (10.3%)	Paracetamol	3 (2.6%)	2 (1.7%)	Ibuprofen	3 (2.6%)	1 (0.9%)	Pregabalin	2 (1.7%)	5 (4.3%)
Diclofenac	2 (1.7%)	1 (0.9%)	Morphine	3 (2.6%)	1 (0.9%)	Naproxen	3 (2.6%)	1 (0.9%)	Oxycodone	2 (1.7%)	4 (3.4%)
Ketorolac	1 (0.9%)	2 (1.7%)	Lidocaine	2 (1.7%)	3 (2.6%)	Paracetamol	2 (1.7%)	3 (2.6%)	Diclofenac	2 (1.7%)	
Celecoxib	1 (0.9%)	2 (1.7%)	Naproxene	2 (1.7%)	2 (1.7%)	Other opioids	2 (1.7%)	2 (1.7%)	Hydromorphone	2 (1.7%)	
Pregabalin	1 (0.9%)	1 (0.9%	Oxycodone	2 (1.7%)	2 (1.7%)	Pregabalin	1 (0.9%)	2 (1.7%)	Lidocaine	2 (1.7%)	
Morphine	1 (0.9%)	1 (0.9%	Diclofenac	2 (1.7%)		Nortriptyline	1 (0.9%)	1 (0.9%)	Ketamine	1 (0.9%)	5 (4.3%)
Nortriptyline	1 (0.9%)		Other NSAID	1 (0.9%)	1 (0.9%)	Hydromorphone	1 (0.9%)	1 (0.9%)	Nortriptyline	1 (0.9%)	2 (1.7%)
SNRIs	1 (0.9%)		Other opioids	1 (0.9%)	1 (0.9%)	Etoricoxib	1 (0.9%)		Other NSAID	1 (0.9%)	1 (0.9%)
Tapentadol	1 (0.9%)		Ketorolac	1 (0.9%)		SNRIs	1 (0.9%)		Fentanyl	1 (0.9%)	1 (0.9%)
Oxycodone	1 (0.9%)		Other COX2-inhib	1 (0.9%)		Buprenorphine	1 (0.9%)		Other opioids	1 (0.9%)	1 (0.9%)
Fentanyl	1 (0.9%)		Hydromorphone	1 (0.9%)		Diclofenac	2 (1.7%)		Celecoxib	2 (1.7%)	
Other NSAID	2 (1.7%)		Celecoxib	3 (2.6%)		Celecoxib	2 (1.7%)		SNRIs	2 (1.7%)	
Other opioids	2 (1.7%)		SNRIs	3 (2.6%)		Fentanyl	1 (0.9%)		Ibuprofen	2 (1.7%)	
Ketamine	2 (1.7%)		Carbamazepine	1 (0.9%)		Ketamine	1 (0.9%)		Carbamazepine	1 (0.9%)	
Etoricoxib	1 (0.9%)		Nortriptyline	1 (0.9%)		No alternative	2 (1.7%)		No alternative	1 (0.9%)	4 (3.4%)
Hydromorphone	1 (0.9%)		Tapentadol	1 (0.9%)		None o/t above	2 (1.7%)	1 (0.9%)	None o/t above	2 (1.7%)	1 (0.9%)
None o/t above	1 (0.9%)	2 (1.7%)	Ketamine	1 (0.9%)		No Answer	66 (56.4%)	72 (61.5%)	No Answer	69 (59.0%)	74 (63.2%)
No Answer	64 (54.7%)	66 (56.4%)	No alternative	1 (0.9%)	5 (4.3%)						
			None o/t above	2 (1.7%)	3 (2.6%)						
			No Answer	66 (56.4%)	74 63.2%)						

Abbreviations: cyclooxygenase-2-inhibitor (COX2-inhib); serotonin- and noradrenalin-reuptake inhibitors (SNRIs); non-steroid-anti-inflammatory drug (NSAID).

**Table 7:** Frequency at which practitioners titrate medication.

Titration Schedules Are Used for the Following Drugs (*N* = 117) (More Than One Item Applicable):	
Medication:	Frequency:
Gabapentin	46
Pregabalin	44
Amitriptyline	40
Nortriptyline	26
SNRIs	25
Tramadol	25
Carbamazepine	24
Morphine	21
Oxycodone	20
Fentanyl	17
Ketamine	16
Hydromorphone	10
Buprenorphine	7
Tapentadol	5
Methadone	4
Lidocaine	4
Celecoxib	3
Hydrocodone	3
Capsaicin	3
Ibuprofen	3
Paracetamol	2
Ketorolac	2
Naproxen	1
Diclofenac	1
Etoricoxib	1
Valproic acid	1
Oxcarbazepine	1
Clonidine	1
Other drugs	1
Gabapentin	8

**Table 8:** Gabapentin titration dosages used by practitioners.

Gabapentin Starting Dose (*N* = 117)	No.	(%)	Gabapentin Maintenance Dose (*N* = 117)	No.	(%)	Gabapentin Maximum Dose (*N* = 117)	No.	(%)
5 mg/kg/day	16	13.7	30 mg/kg/day	11	9.4	60 mg/kg/day	7	6.0
10 mg/kg/day	9	7.7	15 mg/kg/day	7	6.0	30 mg/kg/day	5	4.3
2 mg/kg/day	3	2.6	10 mg/kg/day	4	3.4	40 mg/kg/day	4	3.4
15 mg/kg/day	3	2.6	20 mg/kg/day	3	2.6	50 mg/kg/day	4	3.4
3–5 mg/kg/day	2	1.7	5 mg/kg/day	2	1.7	20 mg/kg/day	3	2.6
6 mg/kg/day	2	1.7	10–15 mg/kg/day	2	1.7	35 mg/kg/day	3	2.6
2–5 mg/kg/day	1	0.9	25 mg/kg/day	2	1.7	45 mg/kg/day	3	2.6
3 mg/kg/day	1	0.9	15–20 mg/kg/day	1	0.9	25 mg/kg/day	2	1.7
4 mg/kg/ day	1	0.9	18 mg/kg/day	1	0.9	70 mg/kg/day	2	1.7
5–7 mg/kg/ day	1	0.9	24 mg/kg/ day	1	0.9	72 mg/kg/day	2	1.7
5–10 mg/kg/ day	1	0.9	35 mg/kg/ day	1	0.9	10 mg/kg/day	1	0.9
5–15 mg/kg/ day	1	0.9	36 mg/kg/ day	1	0.9	15 mg/kg/day	1	0.9
10–15 mg/kg/ day	1	0.9	30–40 mg/kg/ day	1	0.9	24 mg/kg/ day	1	0.9
10–30 mg/kg/day	1	0.9	30–60 mg/kg/ day	1	0.9	40–50 mg/kg/ day	1	0.9
100 mg/day	1	0.9	50 mg/kg/ day	1	0.9	100 mg/kg/ day	1	0.9
100–300 mg/ day	1	0.9	300 mg/ day	1	0.9	600 mg/ day	1	0.9
900 mg/day	2	1.7	900–1800 mg/day	1	0.9	1800 mg/ day	2	1.7
Do not use	3	2.6	Other (efficacy, min effective dose, titrated to patient response to symptoms)	5	4.3	1800–3600 mg/day	1	0.9
No answer	67	57.3	Do not use	1	0.9	2400 mg/day	1	0.9
			No answer	70	59.8	Do not use	1	0.9
						No answer	70	59.8

**Table 9:** Non-pharmacologic approaches used by practitioners in case of chronic neuropathic or mixed pain in children.

Non-Pharmacological Approaches	Neuropathic Pain	Mixed Pain
Physiotherapy	44 (37.6%)	44 (37.6%)
Cognitive behavioral therapy	41 (35.0%)	40 (34.2%)
Transcutaneous electric nerve stimulation (TENS)	33 (28.2%)	35 (29.9%)
Hypnosis	17 (14.5%)	18 (15.4%)
Eye movement desensitization and reprocessing (EMDR)	5 (4.3%)	5 (4.3%)
Iontophoresis	1 (0.9%)	2 (1.7%)
Other interventions	16 (13.7%)	18 (15.4%)

## Supplementary Materials

The following are available online at https://www.mdpi.com/2227-9067/7/11/208/s1, Figure S1: Questionnaire concerning the treatment of chronic neuropathic (or mixed) pain in children (the state of affairs).
