# Supplementary material for: Diagnosis and Treatment of Chronic Neuropathic and Mixed Pain in Children and Adolescents: Results of a Survey Study amongst Practitioners
Source: Children (Basel). 2020 Nov 2;7(11):208. doi: 10.3390/children7110208 (PMC7694093; doi:10.3390/children7110208)
Supplement: Supplementary file 1 [file children-07-00208-s001.pdf]

Supplementary Materials:

Figure S1. Questionnaire concerning the treatment of chronic neuropathic (or mixed) pain in children (the state of affairs).

Information concerning the practitioner:

(Your age is:) __________ years

(Sex:) ☐ F ☐ M

Your profession:

- -general practitioner/family doctor ☐
- -pediatrician ☐
- -pediatric oncologist ☐
- -anesthesiologist ☐
- -pediatric anesthesiologist ☐
- -pediatric anesthesiologist painspecialist ☐
- -neurologist ☐
- -pediatric neurologist ☐
- -rehabilitation specialist ☐
- -clinical pharmacologist ☐
- -other: ☐: ________________________________

You work in:

- -general practice ☐
- -public hospital ☐
- -private hospital ☐
- -academic hospital ☐
- -pediatric hospital (including academic pediatric hospital) ☐
- -rehabilitation clinic ☐
- -other: ☐ :________________________

-Location of practice (name of hospital): ____

____

Country of practice:_______________________________________

How do you define **<u>"chronic pain"</u>** in your practice?

- ☐ Pain lasting more than 3 months
- ☐ Pain which persists past the time of normal healing
- ☐ Other: ___________________________________

-In your hospital, who are involved in treating children with all causes of chronic pain ? (more options possible)

(pediatric) anesthesiologist ☐

(pediatric) anesthesiologist-pain specialist ☐

pediatrician ☐

pediatric oncologist ☐

pediatric neurologist ☐

rehabilitation specialist ☐

specialized pain nurse ☐

psychologist ☐

physiotherapist ☐

other: ☐ :___________________________________

-Is there a dedicated "pain team"? ☐ Yes ☐ No

-How much time (%) of your working week do you treat children with chronic pain (pain lasting more

than 3 months) every week ? _________%

-On average where do you treat children with chronic pain ?

Clinic _________%

In a special policlinic or outdoor department _________%

-On average how many patients in each age group do you see in a year with chronic (= continuous or recurrent pain of more than 3 months) neuropathic or mixed pain ?

3 months - < 3 years: ___________

3 years - 7 years: _______________

8 years – 18 years: _____________

-Among patients in each age group what is the distribution between chronic neuropathic and mixed pain?

	Neuropathic pain	***Mixed pain***
3-months - < 3 years:	___________%	___________%
3 years - 7 years:	___________%	___________%
8 years – 18 years:	___________ %	___________%

-In your practice, what is the usual frequency of clinical visits for neuropathic pain evaluation?

i. ☐ every 4 weeks

ii. ☐ every 6 weeks

iii. ☐ every 3 months

iv. ☐ every 6 months

v. ☐ upon request

vi. ☐ other *please specify* :____

Assessment and diagnosis:

-How do you diagnose neuropathic or mixed pain in children ? (more options possible)

based on history ☐

based on physical examination ☐

based on underlying disease ☐

based on treatment for another disease ☐

based on diagnostic tests ☐

-do you use a specially developed questionnaire to establish the diagnosis ?

DN4 Douleur Neuropathique en 4 questions) ☐

LANSS (Leeds Assessment of Neuropathic Symptoms and Signs) ☐

NPQ (Neuropathic Pain Questionnaire) ☐

Pain Detect ☐

ID-Pain (Identify Pain) ☐

-do you use an age-specific validated pain scale to evaluate pain intensity ?

☐ Yes ☐ No

If so, which one ?: ____

-do you use a scale for physical functioning or quality of life (e.g Ped'sQL) ?

☐ Yes ☐ No

If so, which one ?: ____

-do you use the IASP criteria to establish the diagnosis of neuropathic pain ?

"*pain initiated or caused by a primary lesion or dysfunction in the nervous system*"?

☐ Yes ☐ No

-do you use additional diagnostics to establish the diagnosis: neuropathic pain? (more than one option possible)

QST (Quantitative Sensory Testing) measurement	☐
MRI	☐
Nerve biopsy	☐
EMG	☐

-At your practice, which type of underlying clinical condition(s) are the most frequent among patients with neuropathic or mixed pain?
Please select all applicable items:

i. ☐ Phantom limb pain

ii. ☐ Spinal cord injury

iii. ☐ Trauma and postoperative neuropathic pain

iv. ☐ Burns

v. ☐ Effects of cancer diseases processes and treatment

vi. ☐ Autoimmune, degenerative or inflammatory neuropathies (Guillan-Barré syndrome, Charcot-Marie-Tooth disease, Multiple Sclerosis…)

vii. ☐ Infections (eg HIV infections, toxic effects of anti-retroviral therapy, post-herpetic neuralgia)

viii. ☐ Toxic and metabolic neuropathies (mercury, lead, mitochondrial disorders…)

ix. ☐ Hereditary neurodegenerative disorders (eg Fabry disease)

x. ☐ Complex regional pain syndrome

xi. ☐ Erythromelalgia

xii. ☐ Other *please specify* :____

Treatment:

-Is there a written standard protocol regarding the management of neuropathic or mixed pain in your practice?

☐ Yes ☐ No

-Which medication do you use for the **initial** management of **Moderate** (painscore 4-6) neuropathic or mixed pain ?
Please select all applicable items by giving them a number in the order of choice:

	Neuropathic pain	***Mixed pain***	***Not available in my country***
Paracetamol:	☐ Paracetamol	☐ Paracetamol	☐
NSAID's:	☐ Ibuprofen	☐ Ibuprofen	☐
	☐ Diclofenac	☐ Diclofenac	☐
	☐ Other: ______________	☐ Other: _____________	
COX2-inhibitors:	☐ Celecoxib	☐ Celecoxib	☐
	☐ Etoricoxib	☐ Etoricoxib	☐
	☐ Other: ______________	☐ Other: _____________	
Anticonvulsants:	☐ Gabapentin	☐ Gabapentin	☐
	☐ Carbamazepine	☐ Carbamazepine	☐
	☐ Pregabalin	☐ Pregabalin	☐
Antidepressants:	☐ Amitriptyline	☐ Amitriptyline	☐
	☐ Nortriptyline	☐ Nortriptyline	☐
	☐ SNRIs:______________	☐ SNRIs:______________	☐
Opioids	☐ Tramadol	☐ Tramadol	☐
	☐ Tapentadol	☐ Tapentadol	☐
	☐ Morphine	☐ Morphine	☐
	☐ Oxycodone	☐ Oxycodone	☐
	☐ Fentanyl	☐ Fentanyl	☐
	☐ Buprenorphine	☐ Buprenorphine	☐
	☐ Other: _____________	☐ Other: ______________	
Anesthetics:	☐ Ketamine	☐ Ketamine	☐
	☐ Other: _____________	☐ Other: ______________	

Topical medication:	☐ Lidocaine	☐ Lidocaine	☐
	☐ Capsaicine	☐ Capsaicine	☐

Comments: ____

____

____

____
__

-Which medication do you use for **the initial** management of **Severe** (painscore >7) neuropathic or mixed pain ?
Please select all applicable items by giving them a number in the order of choice:

	Neuropathic pain	***Mixed pain***	***Not available in my country***
Paracetamol:	☐ Paracetamol	☐ Paracetamol	☐
NSAID's:	☐ Ibuprofen	☐ Ibuprofen	☐
	☐ Diclofenac	☐ Diclofenac	☐
	☐ Other: _______________	☐ Other: ______________	
COX2-inhibitors:	☐ Celecoxib	☐ Celecoxib	☐
	☐ Etoricoxib	☐ Etoricoxib	☐
	☐ Other: _______________	☐ Other: ______________	
Anticonvulsants:	☐ Gabapentin	☐ Gabapentin	☐
	☐ Carbamazepine	☐ Carbamazepine	☐
	☐ Pregabalin	☐ Pregabalin	☐
Antidepressants:	☐ Amitriptyline	☐ Amitriptyline	☐
	☐ Nortriptyline	☐ Nortriptyline	☐
	☐ SNRIs:_______________	☐ SNRIs:_______________	☐
Opioids	☐ Tramadol	☐ Tramadol	☐
	☐ Tapentadol	☐ Tapentadol	☐
	☐ Morphine	☐ Morphine	☐
	☐ Oxycodone	☐ Oxycodone	☐
	☐ Fentanyl	☐ Fentanyl	☐
	☐ Buprenorphine	☐ Buprenorphine	☐
	☐ Other: ______________	☐ Other: _______________	
Anesthetics:	☐ Ketamine	☐ Ketamine	☐
	☐ Other: ______________	☐ Other: _______________	

Topical medication: ☐ Lidocaine ☐ Lidocaine ☐

☐ Capsaicine ☐ Capsaicine ☐

Comments: ____

____

____

____

__

-Which medication do you **for second-line** therapy for **Moderate** neuropathic or mixed pain when first-line therapy is not sufficient (Add on) ?

Please select all applicable items by giving them a number in the order of choice:

	Neuropathic pain	***Mixed pain***	***Not available in my country***
Paracetamol:	☐ Paracetamol	☐ Paracetamol	☐
NSAID's:	☐ Ibuprofen	☐ Ibuprofen	☐
	☐ Diclofenac	☐ Diclofenac	☐
	☐ Other: _______________	☐ Other: ______________	
COX2-inhibitors:	☐ Celecoxib	☐ Celecoxib	☐
	☐ Etoricoxib	☐ Etoricoxib	☐
	☐ Other: _______________	☐ Other: ______________	
Anticonvulsants:	☐ Gabapentin	☐ Gabapentin	☐
	☐ Carbamazepine	☐ Carbamazepine	☐
	☐ Pregabalin	☐ Pregabalin	☐
Antidepressants:	☐ Amitriptyline	☐ Amitriptyline	☐
	☐ Nortriptyline	☐ Nortriptyline	☐
	☐ SNRIs:_______________	☐ SNRIs:_______________	☐
Opioids	☐ Tramadol	☐ Tramadol	☐
	☐ Tapentadol	☐ Tapentadol	☐
	☐ Morphine	☐ Morphine	☐
	☐ Oxycodone	☐ Oxycodone	☐
	☐ Fentanyl	☐ Fentanyl	☐
	☐ Buprenorphine	☐ Buprenorphine	☐
	☐ Other: ______________	☐ Other: _______________	
Anesthetics:	☐ Ketamine	☐ Ketamine	☐
	☐ Other: ______________	☐ Other: _______________	
Topical medication:	☐ Lidocaine	☐ Lidocaine	☐

☐ Capsaicine ☐ Capsaicine ☐

Comments: ____

____

____

____
__

-Which medication do you use or add if the initial therapy for **Severe** neuropathic or mixed pain is not sufficient (Add on) ?
Please select all applicable items by giving them a number in the order of choice:

Neuropathic pain	***Mixed pain***		***Not available in my country***
Paracetamol:	☐ Paracetamol	☐ Paracetamol	☐
NSAID's:	☐ Ibuprofen	☐ Ibuprofen	☐
	☐ Diclofenac	☐ Diclofenac	☐
	☐ Other: _______________	☐ Other: ______________	
COX2-inhibitors:	☐ Celecoxib	☐ Celecoxib	☐
	☐ Etoricoxib	☐ Etoricoxib	☐
	☐ Other: _______________	☐ Other: ______________	
Anticonvulsants:	☐ Gabapentin	☐ Gabapentin	☐
	☐ Carbamazepine	☐ Carbamazepine	☐
	☐ Pregabalin	☐ Pregabalin	☐
Antidepressants:	☐ Amitriptyline	☐ Amitriptyline	☐
	☐ Nortriptyline	☐ Nortriptyline	☐
	☐ SNRIs:_______________	☐ SNRIs:_______________	☐
Opioids	☐ Tramadol	☐ Tramadol	☐
	☐ Tapentadol	☐ Tapentadol	☐
	☐ Morphine	☐ Morphine	☐
	☐ Oxycodone	☐ Oxycodone	☐
	☐ Fentanyl	☐ Fentanyl	☐
	☐ Buprenorphine	☐ Buprenorphine	☐
	☐ Other: ______________	☐ Other: _______________	
Anesthetics:	☐ Ketamine	☐ Ketamine	☐
	☐ Other: ______________	☐ Other: _______________	

Topical medication: ☐ Lidocaine ☐ Lidocaine ☐

☐ Capsaicine ☐ Capsaicine ☐

Comments: ____

____

____

____
__

-Do you use a titration schedule for the medication ?

If yes, for which one ?

Please select all applicable items:

Paracetamol:	☐ Paracetamol	☐ Paracetamol
NSAID's:	☐ Ibuprofen	☐ Ibuprofen
	☐ Diclofenac	☐ Diclofenac
	☐ Other: _______________	☐ Other: ______________
COX2-inhibitors:	☐ Celecoxib	☐ Celecoxib
	☐ Etoricoxib	☐ Etoricoxib
	☐ Other: _______________	☐ Other: ______________
Anticonvulsants:	☐ Gabapentin	☐ Gabapentin
	☐ Carbamazepine	☐ Carbamazepine
	☐ Pregabalin	☐ Pregabalin
Antidepressants:	☐ Amitriptyline	☐ Amitriptyline
	☐ Nortriptyline	☐ Nortriptyline
	☐ SNRIs:_______________	☐ SNRIs:_______________
Opioids	☐ Tramadol	☐ Tramadol
	☐ Tapentadol	☐ Tapentadol
	☐ Morphine	☐ Morphine
	☐ Oxycodone	☐ Oxycodone
	☐ Fentanyl	☐ Fentanyl
	☐ Buprenorphine	☐ Buprenorphine
	☐ Other: ______________	☐ Other: _______________
Anesthetics:	☐ Ketamine	☐ Ketamine
	☐ Other: ______________	☐ Other: _______________
Topical medication:	☐ Lidocaine	☐ Lidocaine

☐ Capsaicine ☐ Capsaicine

Comments: ____

____

____

____

-In case you use Gabapentin as initial medication or second-line(add on) medication what is your initial starting dose, what is your target dose for maintenance and what is your maximum dose ?

Starting dose: ________mg/kg/day

Target dose: ________mg/kg/day

Max dose: ________mg/kg/day

-Once medication is effective, how long do you generally treat the patient before you decide to reduce or stop medication ?

___________ weeks

-In case the initial medication is **not** effective, how long do you generally treat the patient before you decide to add on medication ?

___________ weeks

-In case neither the initial medication nor the add-on medication is effective, how long you treat the patient before you decide to stop the medication ?

___________ weeks

-Would you agree to change your first-line medication for neuropathic or mixed pain treatment to participate in a research protocol concerning the treatment of average or severe chronic neuropathic pain in children ?

☐ Yes, I agree

☐ No, I do not agree to change medication

☐ No, I agree to change medication but this would not be feasible in my practice

Comments: ____

____

__

-In your practice, do you use other (non-pharmacological) approaches for chronic neuropathic or mixed pain?

☐ Yes ☐ No

If yes, then could you please indicate which non-pharmacological approaches are usually privileged in your practice?

Neuropathic pain	***Mixed pain***
☐ Transcutaneous electric nerve stimulation	☐ Transcutaneous electric nerve stimulation
☐ Iontophoresis	☐ Iontophoresis
☐ Cognitive behavioral therapy	☐ Cognitive behavioral therapy
☐ Psychological and occupational therapy	☐ Psychological and occupational therapy
☐ EMDR (Eye Movement Desensitization and Reprocessing)	☐ EMDR
☐ Physiotherapy	☐ Physiotherapy
☐ Hypnosis	☐ Hypnosis
☐ Other: ____________________________	☐ Other: ______________________________
